# Results from a prospective international real-world registry of sutureless aortic bioprosthesis by a minimally invasive approach

**DOI:** 10.3389/fcvm.2026.1812903

**Published:** 2026-04-20

**Authors:** Giovanni Concistrè, Max Baghai, Giuseppe Santarpino, Alistair Royse, Mattia Glauber, Marco Solinas

**Affiliations:** 1 Department of Adult Cardiac Surgery, G Pasquinucci Heart Hospital, Massa, Italy; 2 Department of Cardiothoracic Surgery, King’s College Hospital NHS Foundation Trust, London, United Kingdom; 3 Department of Cardiac Surgery, Città di Lecce Hospital, GVM Care & Research, Lecce, Italy; 4 Department of Cardiothoracic Surgery, The Royal Melbourne Hospital, Parkville, VIC, Australia; 5 Department of Cardiac Surgery, Gruppo San Donato, Milano, Italy

**Keywords:** aortic stenosis, aortic valve replacement, minimally invasive procedures, real-world evidence, sutureless valves

## Abstract

**Introduction:**

The aim of this study was to report clinical and hemodynamic results from a real-world registry of aortic valve replacement (AVR) with the Perceval sutureless bioprosthesis, comparing mini-sternotomy (MS) versus mini-thoracotomy (MT) approach.

**Methods:**

This prospective international registry enrolled 1,652 patients across 55 institutions between 2011 and 2021. Patients undergoing isolated AVR by minimally invasive cardiac surgery approaches were analyzed. Preoperative covariates were adjusted using 1:1 propensity score matching, reaching a final cohort of 261 patients for each approach.

**Results:**

Isolated AVR via minimally invasive approaches was performed in 710 patients—406 in MS and 304 in MT. After matching, the baseline characteristics were similar between the two groups, except for the preoperative NYHA class distribution. MT was associated with shorter intensive care unit and hospital stays (*p* = <0.001 and *p* = 0.050, respectively), but with higher cross-clamp and cardiopulmonary bypass times compared to MS (<0.001). Within 30 days, one cardiovascular death occurred in the MS group, while 4 (1.5%) reinterventions were reported in the MT group. Pacemaker implantation was required in 5 (1.9%) patients in the MS group and 14 (5.4%) patients in the MT group, with no statistically significant difference. In the matched cohort, survival probability for late events showed no difference between surgical approaches. Mean pressure gradients remained stable during follow-up, with no difference between the groups.

**Discussion:**

Our propensity-matched analysis demonstrates that the use of Perceval in minimally invasive approaches is associated with low perioperative complication rates. Sutureless implanted in MT has lower intensive care and in-hospital stay without significant differences in long-term clinical and echocardiographic outcomes.

## Introduction

Aortic valve stenosis is the most frequent cardiac valve disease requiring surgical intervention to improve patient quality of life and survival. Over the last decade, the therapeutic alternatives for patients with aortic valve stenosis have expanded thanks to the developments and promising clinical results of transcatheter technologies. However, surgical aortic valve replacement (AVR) remains the procedure of choice in several clinical settings (1, 2).

The Perceval sutureless valve (Corcym, Saluggia, Italy) was introduced into clinical practice in 2007 as an alternative option for cardiac surgeons seeking a simple and rapid aortic valve replacement (AVR) procedure. Its sutureless design facilitates minimally invasive AVR, reducing operating time and mitigating the complications associated with prolonged surgery—an issue commonly encountered when attempting to minimize surgical incision size. Several studies have reported excellent clinical and echocardiographic outcomes in patients implanted with the Perceval valve, both in early and mid-to-long-term follow-up (3–8). Results in different minimally invasive settings have been also published by several groups, underlining the versatility of the Perceval technology (9). Nevertheless, these studies did not provide comparisons of outcomes between different minimally invasive techniques in real-world multicentric cohorts.

The aim of this paper was to report the clinical and hemodynamic results of a subanalysis from the largest prospective real-world registry of the Perceval valve (8), focusing on isolated minimally invasive AVR and comparing outcomes by surgical approaches.

## Materials and methods

The data underlying this article will be shared upon reasonable request to the corresponding author.

The study was registered as SURE-AVR (Sorin Universal Registry on Aortic Valve Replacement) (NCT02679404). The SURE-AVR registry is a real-world, prospective, international, multicenter, observational registry designed to collect safety and performance data on CORCYM aortic devices. Ethics committee and/or institutional review board approval was obtained as required by local regulations. The registry was conducted in compliance with International Conference on Harmonization guidelines and Good Clinical Practice. All enrolled patients provided informed consent for study participation and publication of results as per local regulations.

Preoperative, procedural, and post-implant follow-up clinical and echocardiographic data were collected for all patients and entered into an electronic case report form. Follow-up visits were performed according to the standard of care at each site (telephone call, referring physician, or clinical visit) at 1 year and annually up to 5 years, with follow-up at 7 years in selected centers. Seventy-three centers enrolled patients in a sequential and prospective manner across 18 countries (Europe, Canada, USA, and Australia) in the overall SURE-AVR study cohort on different CORCYM aortic devices. Among these, 55 sites included patients undergoing implantation of Perceval S and Perceval Plus valves (1,652 patients between March 2011 and June 2021). No specific inclusion and exclusion criteria were applied other than the indications and contraindications specified in the “instructions for use” of the Perceval valve, as the study was based on real-world concepts.

This analysis focused on isolated AVR patients who received Perceval S or Perceval Plus valve in minimally invasive settings, comparing mini-sternotomy (MS) versus mini-thoracotomy (MT) approaches.

### Study device

The Perceval valve is a self-expanding, sutureless, surgical aortic biological valve indicated for the replacement of diseased native aortic heart valves or malfunctioning prostheses. This bioprosthesis has a functional component, comprising bovine pericardium, stabilized in buffered glutaraldehyde solution, and a super-elastic metal alloy stent, which has the dual role of providing valve support and anchoring to the aortic root with no permanent sutures. Perceval has a collapsible property, due to which—before implantation—the diameter of the prosthesis is reduced through a collapsing tool for loading the valve in a delivery system. The valve is then positioned and released in the aortic root and subsequently post-dilated using a dedicated balloon catheter. The device is available in four sizes (small, medium, large, and extra-large), covering annular diameters ranging from 19 to 27 mm. Both variants of the Perceval valve—Perceval S and Perceval PLUS (more recently introduced on clinical use and featuring an advanced tissue treatment, FREE)—were implanted at the participating centers. Patients underwent implantation through minimally invasive cardiac surgery approaches, either MS or right anterior MT. The choice of surgical approach was based on surgeon preference after assessment of technical feasibility.

Clinical success was defined as successful valve implantation without the occurrence of major adverse events by the time of hospital discharge. Investigator-reported major adverse events were defined as death (all-cause, cardiovascular, non-cardiovascular), stroke, and reintervention (surgery or any other cardiac invasive therapy). Serious valve-related adverse events included bleeding, thromboembolism, valve thrombosis, endocarditis, non-structural dysfunction, and structural valve deterioration. Severity of valve dysfunction was classified as mild (grade 1+), moderate (grade 2+), moderate to severe (grade 3+), or severe (grade 4+). Echocardiographic data were collected as per standard of care at each center.

### Statistical analysis

**Figure 1:**
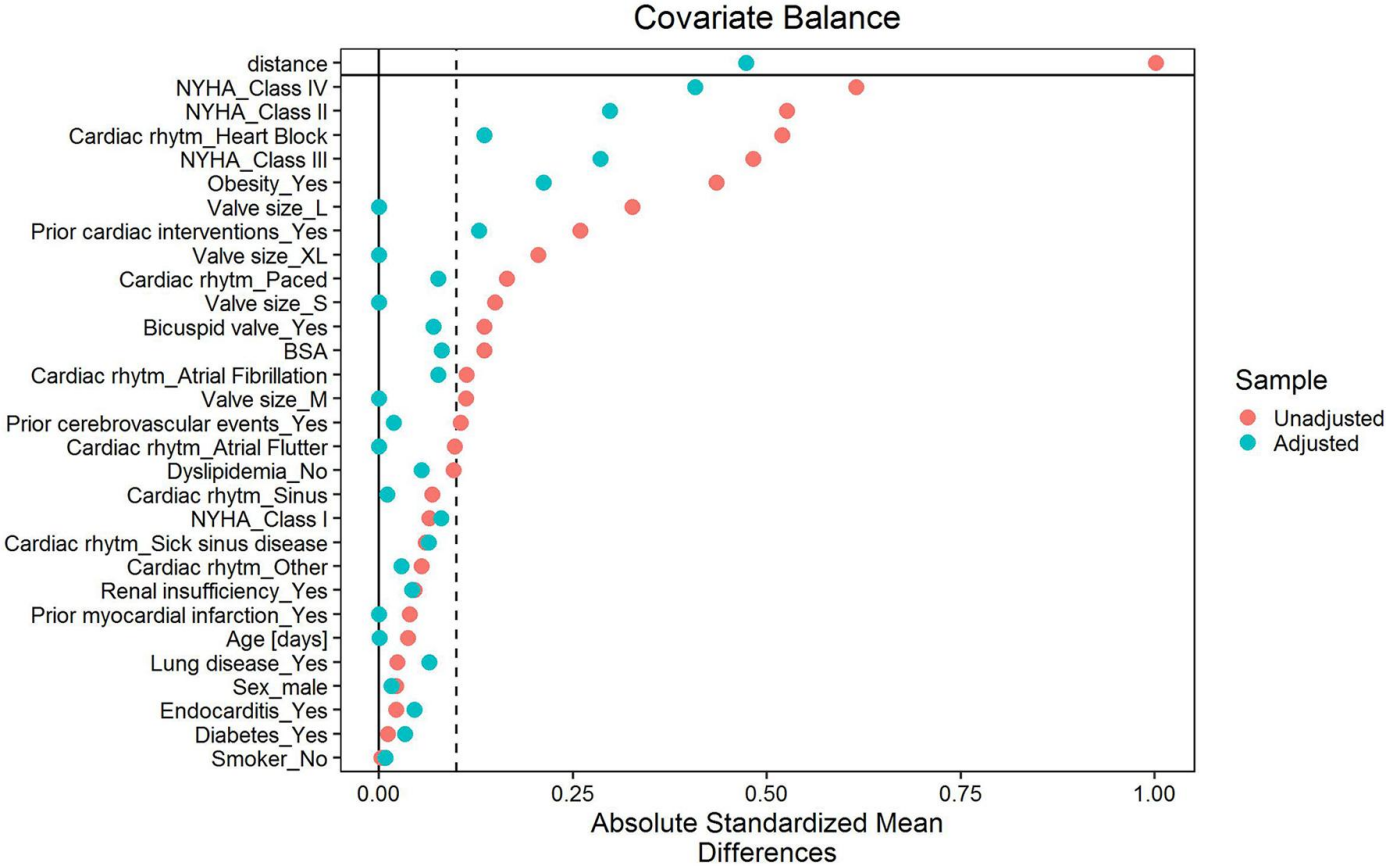
Covariate balance pre-/post-propensity score matching.

To reduce bias in comparing the two approaches, the study groups were matched. A propensity score (PS) was calculated via logistic regression, with numerous matching algorithms tested (nearest-neighbors matching with/without replacement and with/without calipers, full matching with/without calipers, optimal matching, exact matching, genetic matching). The final method selected was optimal matching with exact matching for valve size and a 1:1 matching ratio (MS vs. MT). The best method was defined as the one leading to the best balance (i.e., standardized mean differences <0.1) between the covariates of interest and the highest number of subjects included in matched patients. As shown in Figure 1, the following variables were included in the PS model: age, sex, BSA, diabetes, endocarditis, stroke, lung disease, valve size, smoking status, dyslipidemia, obesity, previous myocardial infarction, renal insufficiency, bicuspid valve, prior cardiac interventions, cardiac rhythm, and NYHA class. Variables were described as mean ± standard deviation or median (quartile Q1, Q3; range) for quantitative variables, and as number (%) for qualitative variables. Outcomes are reported as descriptive statistics. Comparisons between MS and MT were evaluated using independent *t*-tests (if normality assumption was met) or Mann–Whitney *U*-tests (if the normality assumption was not met) for quantitative variables, and Fisher’s exact test for qualitative variables. If Fisher's exact test was computationally complex, the Monte Carlo approximation was used. Normality assessment was conducted via the Shapiro–Wilk test.

The rates of early (defined as occurring up to 30 days) and late (>30 days) adverse events were calculated as the total number of events divided by the total number of patients. The proportion of patients with early and late adverse events was calculated as the total number of patients with the event divided by the total number of patients; if a patient experienced more than one early or late adverse event, they were counted only once as early and once as late. Linearized complication rates [and two-sided 95% confidence intervals (CIs)] were calculated as the number of late events (>30 days) divided by the number of late patient-years.

Multivariable linear regression models and multivariable logistic models were built according to the nature of dependent variables to quantify the effect of the different minimally invasive surgical techniques on the endpoints, while adjusting for the covariates that remained unbalanced even after the matching process.

Late deaths were modeled via stratified log-rank tests and multivariable Cox regression, adjusting for the covariates that remained unbalanced even after the matching process.

Valve-related reinterventions, strokes, and reinterventions due to SVD were modeled via stratified log-rank tests and stratified multivariable competing-risk regressions (Fine and Gray approach), adjusting for the covariates that remained unbalanced even after the matching process, with death included as a competing event. If convergence issues occurred, unstratified competing risk regressions were performed.

A *p*-value below 0.05 was considered statistically significant. All analyses were performed in R software, version 4.3.2 [R Core Team (2023). R Foundation for Statistical Computing, Vienna, Austria.], using the following packages: tidyverse, tableone, MatchIt, lme4, surviva, and cmprisk.

## Results

A total of 1,652 patients implanted with a Perceval S or Perceval PLUS sutureless valve were prospectively enrolled in the SURE-AVR registry between March 2011 and June 2021, as previously described (10). In the overall cohort, mean age was 75.3 ± 7.0 years (53.9% female); mean EuroSCORE II was 4.1 ± 6.3. Isolated AVR via a minimally invasive approach was performed in 710 patients—406 in MS and 304 in MT. The 1:1 propensity-matched analysis resulted in two final cohorts of 261 patients each. After matching, the two groups presented balanced baseline characteristics, except for previous cardiac procedures, preoperative NYHA class distribution, cardiac rhythm, and obesity (Figure 1). Baseline clinical characteristics of the unmatched and matched cohort are reported in Table 1. Exact matching was performed for valve size, with size L being the most used (*N* = 115, 44.1%) (Table 2).

**Table 1:** Baseline characteristics.

VARIABLES	Unmatched				Matched			
	Mini- sternotomy	Mini- thoracotomy	*P* value	SMD	Mini- sternotomy	Mini- thoracotomy	*p*-Value	SMD
	*N* = 406	*N* = 304			*N* = 261	*N* = 261		
Age (years)								
*n*	406	304	0.297*	0.058	261	261	0.854*	0.001
mean (SD)	75.26 (6.51)	75.64 (6.74)			75.68 (6.62)	75.68 (6.87)		
Missing	0	0			0	0		
Female, *n* (%)	237 (58.4)	187 (61.5)	0.439	0.064	159 (60.9)	161 (61.7)	0.928	0.016
Dyslipidemia, *n* (%)	226 (56.4)	179 (60.7)	0.276	0.088	150 (57.5)	157 (60.2)	0.594	0.055
Diabetes, *n* (%)	117 (29.2)	88 (29.9)	0.866	0.017	78 (29.9)	74 (28.4)	0.773	0.034
Chronic lung disease, *n* (%)	61 (15.1)	45 (14.8)	1.000	0.007	43 (16.5)	37 (14.2)	0.544	0.064
Renal insufficiency, *n* (%)	36 (8.9)	24 (7.9)	0.684	0.035	24 (9.2)	21 (8.0)	0.755	0.041
Peripheral vascular disease, *n* (%)	20 (5.9)	8 (10.1)	0.207	0.158	12 (5.6)	4 (6.2)	0.767	0.028
Previous cardiac procedures, *n* (%)	38 (9.4)	55 (18.1)	0.001	0.253	25 (9.6)	38 (14.6)	0.106	0.153
Previous myocardial infarction, *n* (%)	19 (4.7)	17 (5.6)	0.608	0.040	13 (5.0)	13 (5.0)	1.000	<0.001
Previous cerebrovascular events, *n* (%)	26 (6.4)	12 (3.9)	0.178	0.112	13 (5.0)	12 (4.6)	1.000	0.018
Left ventricular ejection fraction								
*n*	340	78	0.519*	0.068	213	63	0.948*	0.005
Mean (SD)	58.76 (9.80)	59.37 (8.06)			59.14 (8.97)	59.10 (8.39)		
Missing	66	226			48	198		
NYHA class I, *n* (%)	18 (4.7)	19 (6.5)	<0.001	0.570	12 (4.6)	17 (6.5)	<0.001	0.372
NYHA class II, *n* (%)	169 (43.9)	198 (67.3)			140 (53.6)	176 (67.4)		
NYHA class III, *n* (%)	175 (45.5)	75 (25.5)			98 (37.5)	66 (25.3)		
NYHA class IV, *n* (%)	23 (6.0)	2 (0.7)			11 (4.2)	2 (0.8)		
Preop sinus rhythm, *n* (%)	325 (80.0)	255 (83.9)	<0.001	0.364	220 (84.3)	221 (84.7)	0.545	0.179
Endocarditis, *n* (%)	2 (0.5)	2 (0.7)	1.000	0.022	1 (0.4)	2 (0.8)	1.000	0.051
Bicuspid valve, *n* (%)	35 (8.6)	35 (11.6)	0.205	0.097	22 (8.4)	28 (10.7)	0.457	0.078

NYHA, New York Heart Association; SD, Standard deviation; SMD, Standardized mean difference.

Numerical data compared using t-test or Mann–Whitney *U*-test, categorical data compared using Fisher’s exact test.

* *p*-value obtained using Mann–Whitney *U*-test.

**Table 2:** Valve size.

Variables	Unmatched		Matched	
	Mini-sternotomy	Mini-thoracotomy	Mini-sternotomy	Mini-thoracotomy
	*N* = 406	*N* = 304	*N* = 261	*N* = 261
Perceval size				
Small, *N* (%)	78 (19.2)	43 (14.1)	40 (15.3)	40 (15.3)
Medium, *N* (%)	132 (32.5)	91 (29.9)	80 (30.7)	80 (30.7)
Large, *N* (%)	128 (31.5)	141 (46.4)	115 (44.1)	115 (44.1)
Extra-large, *N* (%)	68 (16.7)	29 (9.5)	26 (10.0)	26 (10.0)

Successful implantation at the first attempt was achieved in 98.9% of cases in the MS group and 98.1% of cases in the MT group (*p* = 0.724). In the analyzed matched population, receiving a valve via the MT approach resulted in higher cross-clamp and cardiopulmonary bypass (CPB) times compared with MS: median cross-clamp time and CPB were 44 and 64.5 min in the MS group and 55 and 88.5 min in the MT group (Table 3). The differences between the two approaches in terms of procedural times were statistically significant both in the univariable and multivariable analyses (*p*-value < 0.001, in all cases). Conversely, ICU stay was significantly shorter in the MT cohort (*p*-value: ICU-univariable <0.001, ICU- multivariable 0.002) and hospital stay was also shorter in this cohort compared with the MS with a *p*-value of 0.050 in the univariable analysis and <0.001 in the multivariable analysis (Table 3).

**Table 3:** Operative data-matched cohorts.

Variables	Mini-sternotomy (*N* = 261)	Mini-thoracotomy (*N* = 261)	Univariable analysis	Multivariable analysis	
			*p*-Value	Estimated coefficient (95% CI)	*p*-Value
First successful implant, *n* (%)	258 (98.9)	256 (98.1)	0.724		
ICU stay (days), median [IQR]	2.00 [1.00, 2.21]	1.00 [1.00, 2.00]	<0.001*	−0.57 [−0.93, −0.22]	0.002
Hospital stay, median [IQR]	8.65 [7.00, 12.00]	8.00 [7.00, 10.00]	0.050*	−1.06 [−1.96, −0.17]	0.020
Cross-clamp time (min), median [IQR]	44.00 [32.00, 55.00]	55.00 [47.00, 68.50]	<0.001*	14.52 [11.17, 17.87]	<0.001
Pump time (min), median [IQR]	64.50 [47.00, 83.00]	88.50 [75.00, 108.00]	<0.001*	25.46 [20.46, 30.46]	<0.001

ICU, Intensive care unit; IQR, Interquartile range; SD, Standard deviation.

For first successful implant the *P*-value was obtained using Fisher’s exact test. The coefficients and confidence intervals were estimated via multivariable linear regression and refer to the effect of receiving a mini-thoracotomy treatment.

* *p*-Value obtained using Mann–Whitney *U*-test.

Within 30 days, two deaths (0.8%) occurred in the MS group, including one cardiovascular death, while no deaths were reported in the MT group. There were no valve-related reinterventions in the MS group, while four (1.5%) were reported in the MT group. Rates of non-disabling stroke (1.1% and 0.8%), transient ischemic attack (1.1% and 0.8%), bleeding (1.1% and 0.8%), myocardial infarction (0 and 0.4%), intra-prosthetic regurgitation (0.4% and 1.1%), and para-prosthetic regurgitation (0.8% and 0.4%) (≥2) in the MS and MT groups were low. No cases of disabling stroke, thromboembolism, endocarditis, and valve thrombosis were reported. Pacemaker implantation was required in 5 (1.9%) of patients in the MS group and 14 (5.4%) of patients in the MT group. Both the univariable and the multivariable analyses showed no difference between the groups for the early complications (Table 4).

**Table 4:** Early results (≤30 days)-matched cohorts.

Variables	Mini-sternotomy (*N* = 261)	Mini-thoracotomy (*N* = 261)	Mini-sternotomy (*N* = 261)	Mini-thoracotomy (*N* = 261)	Univariable analysis	Multivariable analysis	
	AEs (%AEs on *N*)	AEs (%AEs on *N*)	pts (%pts on *N*)	pts (%pts on *N*)	*p*-Value	Estimated OR (95% CI)	*p*-Value
All deaths, *n* (%)	2 (0.8)	0	2 (0.8)	0	0.499	n.a.	n.a.
Cardiovascular deaths, *n* (%)	1 (0.4)	0	1 (0.4)	0	1	n.a.	n.a.
Valve-related reintervention, *n* (%)	0	4 (1.5)	0	4 (1.5)	0.124	n.a.	n.a.
Reintervention due to SVD, *n* (%)	0	0	0	0	n.a.	n.a.	n.a.
TIA, *n* (%)	3 (1.1)	2 (0.8)	3 (1.1)	2 (0.8)	1	0.58 [0.07, 3.72]	0.566
Disabling stroke, *n* (%)	0	0	0	0	n.a.	n.a.	n.a.
Non-disabling stroke, *n* (%)	3 (1.1)	2 (0.8)	3 (1.1)	2 (0.8)	1	0.56 [0.07, 3.52]	0.536
Bleeding, *n* (%)	3 (1.1)	2 (0.8)	3 (1.1)	2 (0.8)	1	0.55 [0.06, 3.64]	0.534
Thromboembolism, *n* (%)	0	0	0	0	n.a.	n.a.	n.a.
Intra-prosthetic regurgitation (≥2), *n* (%)	1 (0.4)	3 (1.1)	1 (0.4)	3 (1.1)	0.624	3.07 [0.38, 63.52]	0.339
PVL (≥2), *n* (%)	2 (0.8)	1 (0.4)	2 (0.8)	1 (0.4)	1	0.47 [0.02, 5.15]	0.543
Endocarditis, *n* (%)	0	0	0	0	n.a.	n.a.	n.a.
Valve thrombosis, *n* (%)	0	0	0	0	n.a.	n.a.	n.a.
Myocardial infarction, *n* (%)	0	1 (0.4)	0	1 (0.4)	1	n.a.	n.a.
Pacemaker implant, *n* (%)	5 (1.9)	14 (5.4)	5 (1.9)	14 (5.4)	0.059	2.92 [1.05, 9.64]	0.053

n.a., Result not available, as there are 0 events in at least one cohort; OR, odds ratio (mini-thoracotomy/mini-sternotomy), as per multivariable logistic regression; CI, confidence interval for the OR; SVD, structural valve degeneration; TIA, transient ischemic attack; PVL, paravalvular leakage.

Number of AEs is not displayed since it is the same as the number of patients affected by the AEs. *P*-values calculated on pts. Univariable *p*-values were calculated using Fisher’s exact test. Percentages are based on *N*.

**Figure 2:**
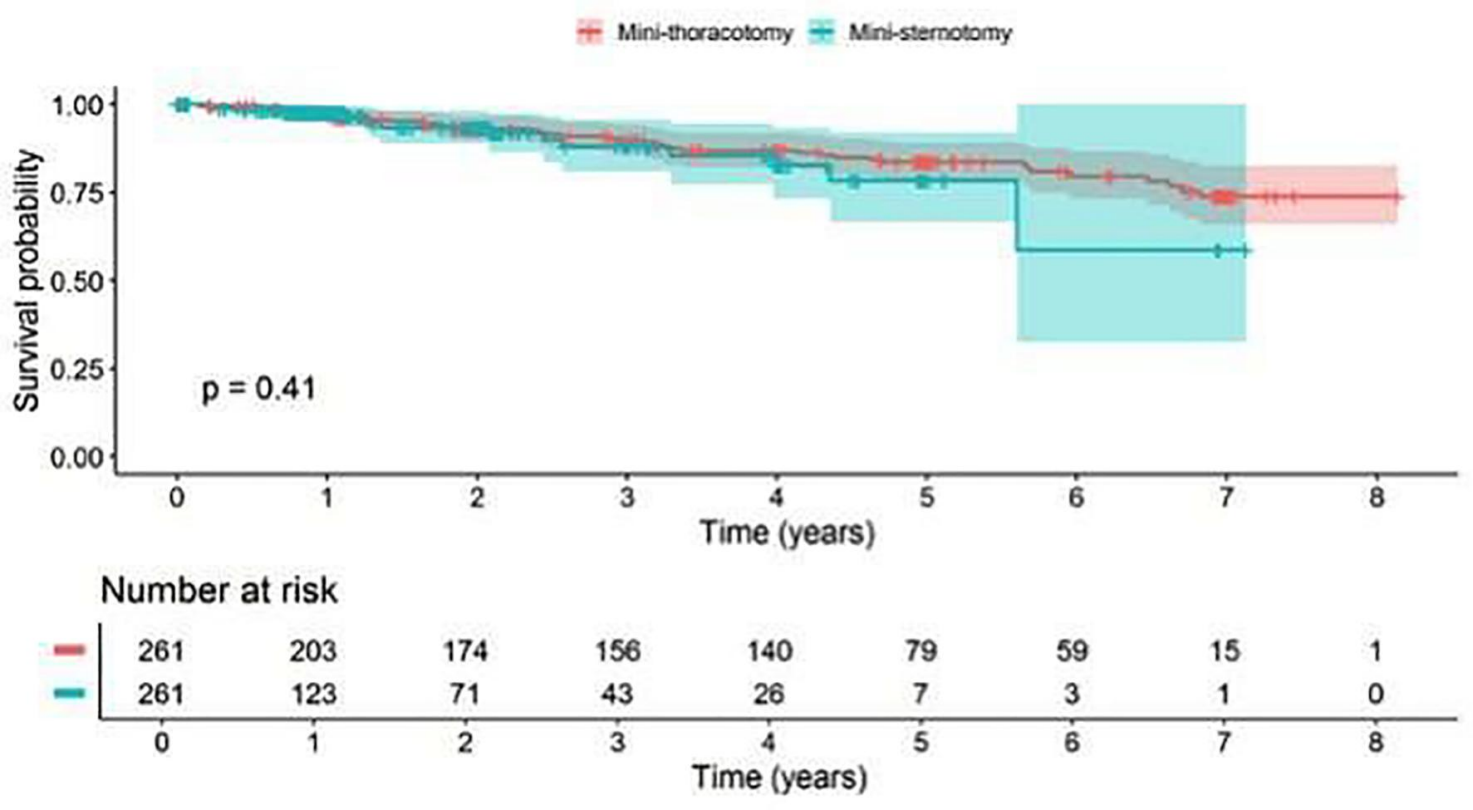
Survival probability on late events (>30 days) by surgical approach-matched cohort.

Cumulative follow-up was 356.8 patient-years for the MS cohort and 963.0 patient-years for the MT one. Survival probability up to 8-year follow-up did not show a statistically significant difference between the matched groups (Figure 2).

Results of the analysis of late outcomes for the matched cohort are displayed in Table 5. Differences in the surgical approaches were highlighted for all deaths (MS 5.7% vs. MT 14.2%, univariable *p*-value: 0.002, multivariable *p*-value < 0.001), but no statistically significant difference was found among the cardiovascular deaths (MS 2.7% vs. MT 6.5%, univariable *p*-value: 0.058, multivariable *p*-value: 0.078). The difference in probability of late valve-related reinterventions in the two groups was statistically significant (MS 0.8% vs. MT 4.6%, univariable *p*-value: 0.012, multivariate *p*-value: 0.009). Reintervention due to structural valve deterioration occurred in eight cases (3.1%) in the MT group, while no cases were reported in the MS group (univariable *p*-value: 0.007). As no event occurred in the MS cohort, building a model was not possible. Structural valve degeneration occurred in eight patients at a mean follow-up of 5.8 ± 1.4 years (range: 2.6–7.3 years). Late bleedings were reported only for the MT group (2.3%), leading to a statistically significant difference against the MS cohort (univariable *p*-value: 0.030); in this case as well, it was not possible to build a model due to the lack of event in the MS group.

**Table 5:** Late results (>30 days)-matched cohorts.

Variables	Mini-sternotomy (356.8 late pt-yrs)	Mini-thoracotomy (963.0 late pt-yrs)	Mini-sternotomy (*N* = 261)	Mini-thoracotomy (*N* = 261)	Univariable analysis	Multivariable analysis	
	*N*—% AEs on 356.8 pts-yrs (2-side 95% CI)	*N*—% AEs on 963.0 pts-yrs (2-side 95% CI)	pts (%pts on *N*)	pts (%pts on *N*)	*p*-Value	Estimated OR [95% CI]	*p*-Value
All deaths, *n* (%)	15–4.2 (2.2–6.4)	37–3.8 (2.7–5.1)	15 (5.7)	37 (14.2)	0.002	3.30 [1.72, 6.70]	<0.001
Cardiovascular deaths, *n* (%)	7–2.0 (0.6–3.6)	17–1.8 (0.9–2.7)	7 (2.7)	17 (6.5)	0.058	2.32 [0.94, 6.29]	0.078
Valve-related reintervention, *n* (%)	2–0.6 (0.0–1.4)	12–1.2 (0.6–2.0)	2 (0.8)	12 (4.6)	0.012	8.52 [2.09, 60.67]	0.009
Reintervention due to SVD, *n* (%)	0	8–0.8 (0.3–1.5)	0	8 (3.1)	0.007	n.a.	n.a.
TIA, *n* (%)	2–0.6 (0.0–1.4)	5–0.5 (0.1–1.0)	2 (0.8)	5 (1.9)	0.450	2.82 [0.57, 20.59]	0.231
Disabling stroke, *n* (%)	2–0.6 (0.0–1.4)	1–0.1 (0.0–0.3)	2 (0.8)	1 (0.4)	1	0.66 [0.03, 7.23]	0.740
Non-disabling stroke, *n* (%)	3–0.8 (0.0–2.0)	5–0.5 (0.1–1.0)	3 (1.1)	5 (1.9)	0.724	1.53 [0.36, 7.81]	0.574
Bleeding, *n* (%)	0	7–0.7 (0.2–1.3)	0	6 (2.3)	0.030	n.a.	n.a.
Thromboembolism, *n* (%)	0	0	0	0	n.a.	n.a.	n.a.
Intra-prosthetic regurgitation (≥2), *n* (%)	2–0.6 (0.0–1.4)	12–1.2 (0.6–2.0)	2 (0.8)	11 (4.2)	0.021	5.75 [1.48, 37.99]	0.026
PVL (≥2), *n* (%)	0	0	0	0	n.a.	n.a.	n.a.
Endocarditis, *n* (%)	2–0.6 (0.0–1.4)	3–0.3 (0.0–0.7)	2 (0.8)	3 (1.1)	1	2.17 [0.30, 23.12]	0.458
Valve thrombosis, *n* (%)	0	0	0	0	n.a.	n.a.	n.a.
Myocardial infarction, *n* (%)	1–0.3 (0.0–0.8)	4–0.4 (0.1–0.8)	1 (0.4)	4 (1.5)	0.373	4.34 [0.60, 87.47]	0.200
Pacemaker implant, *n* (%)	5–1.4 (0.3–2.8)	8–0.8 (0.3–1.5)	5 (1.9)	8 (3.1)	0.576	1.69 [0.51, 6.17]	0.401

n.a., result not available, as there are 0 events in at least one cohort; OR, odds ratio (mini-thoracotomy/mini-sternotomy), as per multivariable logistic regression; CI, confidence interval for the OR; SVD, structural valve degeneration; TIA, transient ischemic attack; PVL, paravalvular leakage.

*P*-values calculated on pts. Univariable and multivariable analyses are performed on *N*. Univariable *p*-values were calculated using Fisher’s exact test.

Percentages are based on patient-years or *N* according to the description on the header.

Intra-prosthetic regurgitation ≥2 occurred more frequently in the MT group compared with the MS group (4.2% vs. 0.8%; univariable *p*-value: 0.021, multivariable *p*-value: 0.026).

No differences were observed between the groups for disabling stroke, non-disabling stroke, transient ischemic attack, endocarditis, myocardial infarction, and pacemaker implantation. No events of thromboembolism, paravalvular leakage (≥2), and valve thrombosis were registered for both groups.

Hemodynamic results are summarized in Table 6. The comparison between the two approaches was evaluated in terms of mean pressure gradient: No difference was found between the two different surgical approaches (univariable analysis), despite fewer echocardiographic assessments being available in the MS cohort. Values remained stable during the follow-up in both groups.

**Table 6:** Hemodynamic data-matched cohorts.

Variables	Mini-sternotomy	Mini-thoracotomy	Univariable analysis
			*p*-Value
Preop	*N* = 241	*N* = 247	
Effective orifice area (cm²)			
Median [IQR]	0.70 [0.59, 0.90]	0.60 [0.50, 0.80]	
Missing	75	66	
Mean gradient (mmHg)			0.035*
Median [IQR]	47.00 [40.00, 58.00]	51.00 [42.00, 60.00]	
Missing	9	6	
Peak gradient (mmHg)			
Median [IQR]	77.90 [65.00, 94.00]	80.00 [66.00, 97.00]	
Missing	15	36	
Discharge	*N* = 183	*N* = 229	
Effective orifice area (cm²)			
Median [IQR]	1.60 [1.40, 2.00]	1.65 [1.18, 2.01]	
Missing	125	221	
Mean gradient (mmHg)			0.067*
Median [IQR]	13.00 [10.00, 16.00]	13.00 [10.75, 17.00]	
Missing	7	1	
Peak gradient (mmHg)			
Median [IQR]	23.00 [18.00, 30.00]	25.00 [20.00, 32.00]	
Missing	18	153	
1 year	*N* = 44	*N* = 125	
Effective orifice area (cm²)			
Median [IQR]	1.50 [1.20, 1.80]	1.55 [1.30, 2.00]	
Missing	23	5	
Mean gradient (mmHg)			0.670*
Median [IQR]	11.00 [7.78, 16.00]	11.00 [8.00, 15.00]	
Missing	0	2	
Peak gradient (mmHg)			
Median [IQR]	19.95 [14.00, 27.00]	19.00 [14.00, 24.00]	
Missing	4	3	
5 years	*N* = 10	*N* = 112	
Effective orifice area (cm²)			
Median [IQR]	1.20 [1.00, 1.70]	1.50 [1.19, 1.80]	
Missing	1	12	
Mean gradient (mmHg)			0.394*
Median [IQR]	12.00 [10.25, 16.50]	11.50 [8.25, 15.00]	
Missing	0	2	
Peak gradient (mmHg)			
Median [IQR]	20.50 [16.25, 28.75]	20.00 [14.00, 24.00]	
Missing	0	7	

IQR, interquartile range.

Univariable *p*-values were calculated using independent t-test or Mann–Whitney *U*-test.

* *p*-Value obtained using Mann–Whitney *U*-test.

## Discussion

This study reports outcomes from a subgroup of patients who underwent AVR with the Perceval S or Perceval PLUS sutureless valves within in SURE-AVR registry, comparing two minimally invasive approaches—MS versus MT. Our analysis demonstrates substantially comparable results between the two approaches with regard to mortality and short- and long-term outcomes. The MT approach showed better outcomes for reduced length of intensive care and in-hospital stays. Results of our analysis showed statistically significant differences in favor of the MS group for late reinterventions due to SVD, but this could be explained by the longer follow-up available for the MT group. Unlike previous published reports, this study was prospective and analyzed the largest multicenter cohort of patients with Perceval bioprosthesis.

The sutureless bioprosthesis introduces an innovative approach for surgical AVR, designed for faster implantation, thereby minimizing CPB and ACC time durations. These advantages extend to all patients, regardless of their risk profile. Consequently, sutureless aortic valve implantation can serve as an alternative treatment option for individuals at high-risk for mortality and morbidity following open-heart surgery. The first clinical outcomes of the Perceval bioprosthesis were documented in 2011 by Flameng and colleagues (10). In a large multicenter cohort study, Fischlein et al. observed low event rates at the 1-year mark in intermediate-risk patients undergoing AVR (3). Moreover, Concistrè et al. evaluated clinical and hemodynamic performance using data from the largest real-world prospective registry of the Perceval valve (8).

The reduced time needed for implantation is a potential advantage of this prosthesis. In the overall cohort of the SURE-AVR study (1,652 patients) (8), overall cross-clamp time was 61.0 ± 29.9 min and pump time was 90.3 ± 42.2 min. In the STS Database, the ACC and CPB times for AVR in full sternotomy were 77.9 and 106.4, respectively. A meta-analytical study showed CPB times of 104.4 min in patients who underwent AVR through minimally invasive access versus 94.0 min for the conventional access group (*p* < 0.00001) (11). In the present analysis, a CPB time of 79.7 ± 30.4 min for all Perceval AVR through minimal access was reported. The MT approach resulted in higher ACC and CPB times compared with the MS group, with a median cross-clamp time and CPB of 44 and 64.5 min in the MS group and 55 and 88.5 min in the MT group, respectively (*p*-value < 0.001). This result is not unexpected as it is well known that MT is more technically demanding due to the limited surgical access and reduced visibility compared to the MS approach. Consequently, MT interventions are related to longer operation times. As minimally invasive AVR has shown longer CPB and ACC times than conventional surgery, we believe that sutureless technology might be the solution for less invasive approaches. The ACC and CPB times reported in our analysis, for the MS and the MT groups, were significantly lower than the previous published experiences on traditional valves (11), supporting this consideration. On the other hand, ICU stay was statistically significantly lower in the MT cohort (*p* 0.002) and hospital stay was also lower in this cohort compared with the MS group, with a *p* < 0.001. The advantages of Perceval implantation in the mini-sternotomy approach have been described by Fischlein et al., highlighting good results with this minimally invasive technique (4). On the other hand, Massa reported the advantages of AVR with sutureless implantation through a right mini-thoracotomy (12). The present analysis confirmed through a large prospectively multicentric cohort that both approaches are technically feasible and lead to good and stable clinical and hemodynamics outcomes.

In our analysis, 30-day cardiovascular death occurred in one patient from the MS group and no patient in the MT group. Early outcomes showed a low rate of neurological events and reinterventions in both groups. In the MT group, at 30 days, two patients experienced valve malpositioning, one patient had a non-structural valve dysfunction, and one patient had incomplete expansion of the stent valve caused by the calcification of the aortic root. At discharge, the mean transvalvular gradient was 13 mmHg in both groups (*p* *=* 0.065). Relative to other studies, we found similar results in terms of paravalvular leakage and hemodynamic performance. Mean transvalvular gradient at 5-year follow-up was similar in both groups—12 mmHg in the MS group and 11.5 mmHg in the MT group (*p* = 0.394). The rate of perioperative pacemaker implantation at 30 days was higher in the MT group (5.4% vs. 1.9%), but not statistically significant. At a maximum follow-up of 8 years, survival probability related to late events in the matched cohort showed no difference by surgical approach. Results of the univariable and multivariable analyses showed statistically significant differences in favor of the MS group for late reinterventions due to structural valve deterioration (SVD). There were eight cases of reintervention due to SVD in the MT group. Seven of these patients underwent transcatheter valve-in-valve implantation, with a safe, feasible, and favorable procedure, and one patient underwent surgical sutured valve replacement. No differences were found for disabling stroke, non-disabling stroke, transient ischemic attack, endocarditis, myocardial infarction, and pacemaker implantation. No events of thromboembolism, paravalvular leakage (≥2), and valve thrombosis were registered for either group. At follow-up, no statistically significant difference was found for cardiovascular deaths. Hemodynamic data were similar in both groups. In terms of mean pressure gradient, no difference was found (univariable analysis), despite the number of available echocardiographic assessments being lower in the MS cohort. Values remained stable during the follow-up in both groups.

## Limitations

Our study shares the inherent limitations of any observational, single-arm, non-randomized study lacking a comparison with other devices. Another limitation is the absence of monitoring and adjudication for adverse events. The number of isolated patients included in the present analysis is lower than the number reported in previous publications related to the SURE-AVR study cohort; this difference is due to the fact that the information on the type of minimally invasive surgical access was not available for all the patients and therefore it was not possible to include them in the present analysis that had the aim to compare mini-sternotomy and mini-thoracotomy approaches. The choice of surgical approach was based on surgeon preference, and details were not collected regarding the surgical procedures performed and the techniques used may have been different among the centers, representing a potential source of bias. Moreover, echocardiographic follow-up was not available for all the patients and not systematically performed since it was done according to the site's routine standard of care. In-hospital and ICU stay can be influenced by institutional practices and discharge criteria, and may be subject to discretionary variation; however, this limitation applies to both the mini-thoracotomy and the mini-sternotomy approaches, and there is a similar distribution of approaches across the various centers of the study. Finally, the maximum follow-up was 8 years and was more limited in the mini-sternotomy group; longer follow-up data should be collected to further prove the effectiveness of this bioprosthesis.

## Conclusions

Our multicenter, real-world experience with the Perceval sutureless valve showed favorable clinical and hemodynamic results at short- and long-term follow-up. Minimally invasive AVR with a sutureless valve overall achieved favorable outcomes. Propensity-matched analysis demonstrated similar results between the two approaches in term of mortality and stroke, with reduced lengths of intensive care and in-hospital stay in the MT group. There were no statistically significant differences in term of long-term clinical and echocardiographic outcomes. Both approaches are valid, and overall there is no approach that demonstrates a clear superiority over the other.

## Data Availability

The raw data supporting the conclusions of this article will be made available by the authors, without undue reservation.
